# Bionanocomposite MIL-100(Fe)/Cellulose as a high-performance adsorbent for the adsorption of methylene blue

**DOI:** 10.1038/s41598-024-65531-1

**Published:** 2024-06-24

**Authors:** Shahla Abbasi, Zahra Nezafat, Shahrzad Javanshir, Behzad Aghabarari

**Affiliations:** 1 Pharmaceutical and Heterocyclic Compounds Research Laboratory, Chemistry Department, Iran University of Science and Technology, Tehran, 16846-13114 Iran; 2 Nanotechnology and Advanced Materials Department, Materials and Energy Research Center, Karaj, 31787-316 Iran

**Keywords:** Methylene blue, Adsorption, Metal–organic framework, MIL-100, Cellulose, MOF, Chemistry, Materials science

## Abstract

World production of dyes is estimated at more than 800,000 t·yr^−1^. The purpose of this research falls within the scope of the choice of an effective, local, and inexpensive adsorbent to remove dyes from wastewater. Adsorptive elimination of dyes by commonly accessible adsorbents is inefficient. The metal–organic frameworks (MOFs) are an important class of porous materials offering exceptional properties as adsorbents by improving separation efficiency compared to existing commercial adsorbents. However, its powder form limits its applications. One way to overcome this problem is to trap them in a flexible matrix to form a hierarchical porous composite. Therefore, in this work, we prepared MIL-100 (Fe) embedded in a cellulose matrix named MIL-100(Fe)/Cell, and used it as an adsorbent of methylene blue (MB) dye. According to the BET analysis, the specific surface area of the synthesized MOF is 294 m^2^/g which is related to the presence of the cellulose as efficient and green support. The structure of this composite is approximately hexagonal. Adsorption was studied as a function of contact time, adsorbent mass and pollutant load (concentration), and pH, and the effect of each of them on absorption efficiency was optimized. The MIL-100(Fe)/Cell was capable of removing 98.94% of MB dye with an initial concentration of 150 mg/L within 10 min at pH = 6.5 and room temperature. The obtained maximum adsorption capacity was 384.615 mg/g. The adsorption isotherm is consistent with the Langmuir models. The mechanism of MB adsorption proceeds through п-п and electrostatic interactions.

## Introduction

Today, more than 6.5 billion human beings have to make do with the same amount of water, which is why the raw material water, for a long time freely available in many parts of the earth, is still seriously threatened. Industrial effluents and pollutants resulting from the intensive use of fertilizers, pesticides, and sanitary, agricultural, and pharmaceutical products are the major causes of environmental pollution. Pollution generated by human activities represents an increasingly worrying threat to humans and ecosystems, which requires effective techniques to separate and remove them from the environment^1–4^. Among the industries that consume large quantities of water, textiles, and dyeing are at the top of the list, and the sectors of dyeing, printing, or finishing of textiles occupy the following positions. These effluents are heavily loaded with acidic or basic dyes, salts, and additives^5–9^. Several complementary treatments are possible such as adsorption or degradation by oxidation^10,11^. One of the most important and common decontamination of water from pollutants is the adsorption method. The adsorption techniques have many advantages such as cost-effectiveness, flexibility and ease of design, simplicity of operation, and insensitivity to poisonous pollutants. In addition, the adsorption does not result in the creation of damaging materials. Also, the adsorption methods usually are environmentally friendly methods for water treatment^12–17^. There are different types of materials that can be used as sorbent. These materials include graphene oxide (GO), zeolites, MOFs, magnetic nanoparticles, natural polymers, etc. One of the important challenges that researchers are dealing with is to make a sorbent that is both easy to synthesize and effective for adsorbing pollutants^18–23^.

Metal–organic frameworks (MOFs) are a unique class of porous crystalline materials, formed by the interconnection of aggregated metal ions with organic molecules bricks. The characteristics of these structures include high specific surface area and porosity, adjustable functional groups, controlled pore size, and a simple preparation process. These features cause MOF materials to have many applications such as photocatalytic degradation, gas storage, drug delivery, sensors, energy conversion, storage, etc*.*^24^. The use of MOFs to adsorb dyes has been developed in recent years^25,26^. MOFs can be prepared with various dimensionalities in addition to diverse sizes and shapes, reliant on the kind of preparation procedure and materials used. There are different organic ligands used for the synthesis of MOFs and these ligands play significant roles in the structure of MOFs as well as in their properties like adsorption performance, and thermal and aquatic stability. In addition, the MOFs can be synthesized with different types of natural polymers like cellulose, chitosan, starch, alginate, etc. This causes various properties to be added to the MOFs and increases their performance by adsorbing pollutants^27–30^.

Cellulose is a polysaccharide mainly produced by plants. The important properties of cellulose are its abundance in nature, cheapness, non-toxicity, biocompatibility, and biodegradability. Due to these features, cellulose is a suitable option for preparing natural supports in the manufacture of heterogeneous catalysts^31,32^. Cellulose is composed of many glucose units which bind together with 1,4-glycosidic bonds. Owing to its units, cellulose has many –OH functional groups which can be simplified and functionalized with different materials^33^. In this research, we synthesized MIL-100(Fe) on cellulose support (MIL-100/Cell) by simple method at ambient temperature using water as a green solvent and used for MB adsorption. The MB was used as the target pollutant because it is broadly applied in industries like pharmaceuticals, food, textile, and printing, and there are large amounts of it in wastewater. In addition, we optimized different factors for the adsorption like initial concentration of the dye, adsorbent doses, pH, and time.

## Results and discussion

### Characterization of MIL-100/Cell

The prepared MIL-100/Cell was analyzed by diverse analytical techniques like FTIR, XRD, SEM, TGA/DTA, BET, EDS, and elemental mapping.

**Figure 1:**
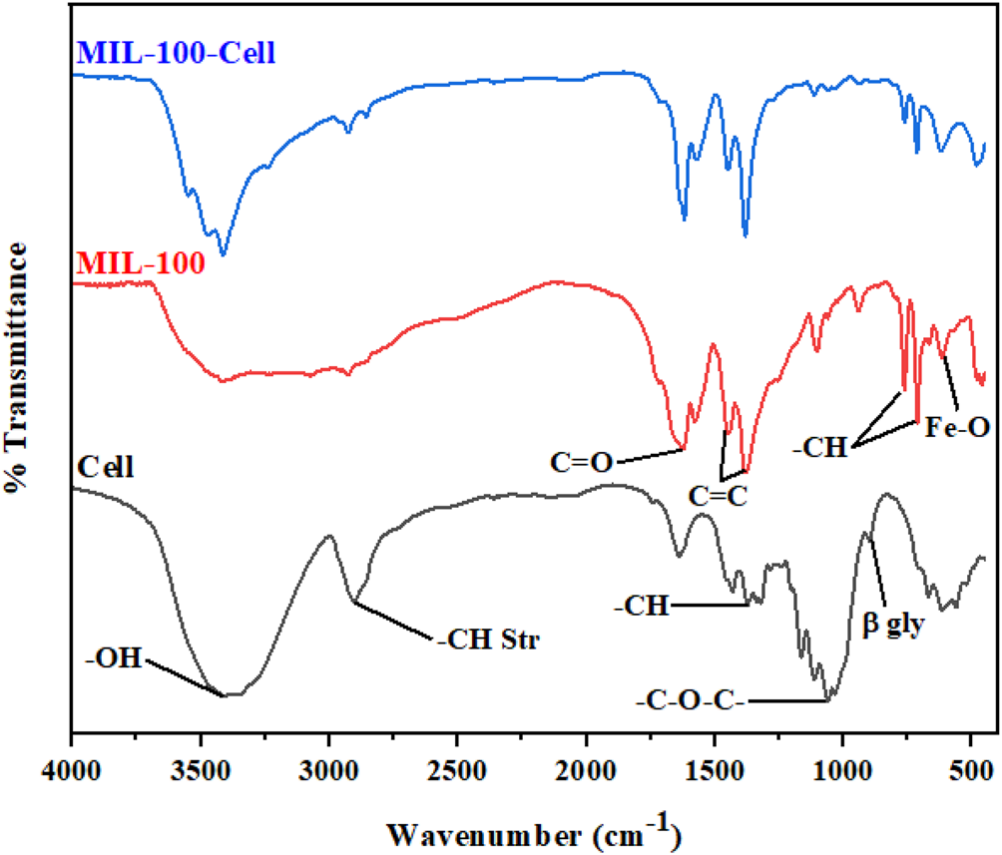
FTIR spectra of the cellulose (Cell), MIL-100, and MIL-100/Cell.

Figure 1 displays the FTIR spectra of cellulose, MIL-100, and MIL-100/Cell. According to the cellulose spectrum, the absorption peaks at 901 and 1063 cm^−1^ correspond to the β-glycosidic bond and –C–O–C– pyranose ring, respectively. The absorption bands at 1374 and 2900 cm^−1^ are correlated to the stretching and bending vibrations of –CH of glucose units of cellulose. Also, the absorption peak at 3400 cm^−1^ is related to the stretching vibrations of –OH in the structure of cellulose^34^. In the MIL-100 spectrum, some new adsorption peaks confirmed the formation of the MOF.

For the synthesis of MIL-100, we used benzene-1,3,5-tricarboxylic acid (BTC) and FeCl_2_.4H_2_O. In the chemical structure of the BTC, there are benzene rings and C=O functional groups. The absorption peak related to the C=O bond at 1620 cm^−1^, the C=C bond of the benzene ring at 1379 cm^−1^ and 1446, and the –CH of benzene at 712 cm^−1^ and 763 are confirmed. In addition, because of the presence of the iron salt, the Fe–O bonds are created among BTC and iron salt, so the Fe–O bond at 615 cm^−1^ is detected. After placing MIL-100 on the cellulose substrate, some peaks change. According to the FTIR of the MIL-100/Cell, all of the mentioned peaks which are presented on cellulose and MIL-100 are in the FTIR of the MIL-100/Cell too.

**Figure 2:**
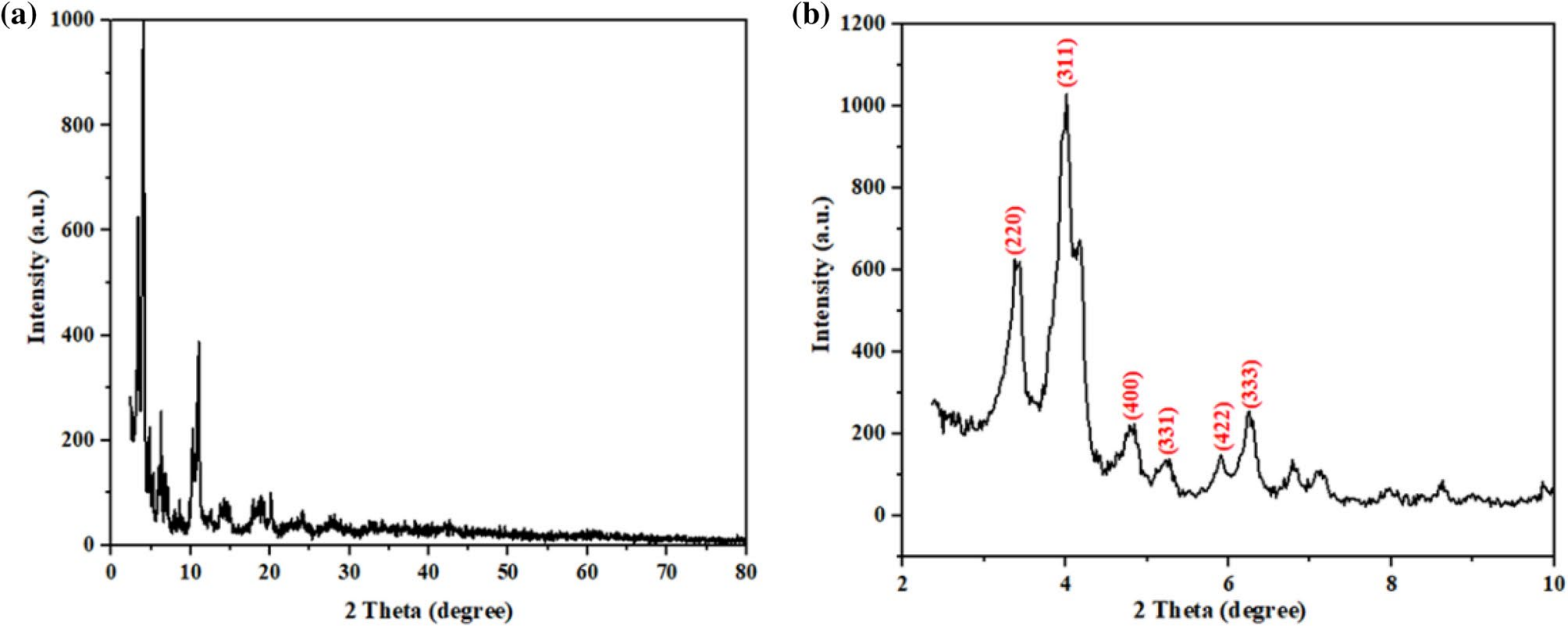
(**a**) XRD and (**b**) expand XRD pattern of the MIL-100/Cell.

The crystal structure of the synthesized MIL-100/Cell was confirmed by XRD analysis (Fig. 2a and b). According to the Fig. 2b, the observed peaks at 2θ = 3.4, 4, 4.8, 5.2, 5.9, and 6.2 correspond to the lattice planes [220], [311], [400], [331], [422] and [333] respectively which is identical to the reported patterns^35^.

**Figure 3:**
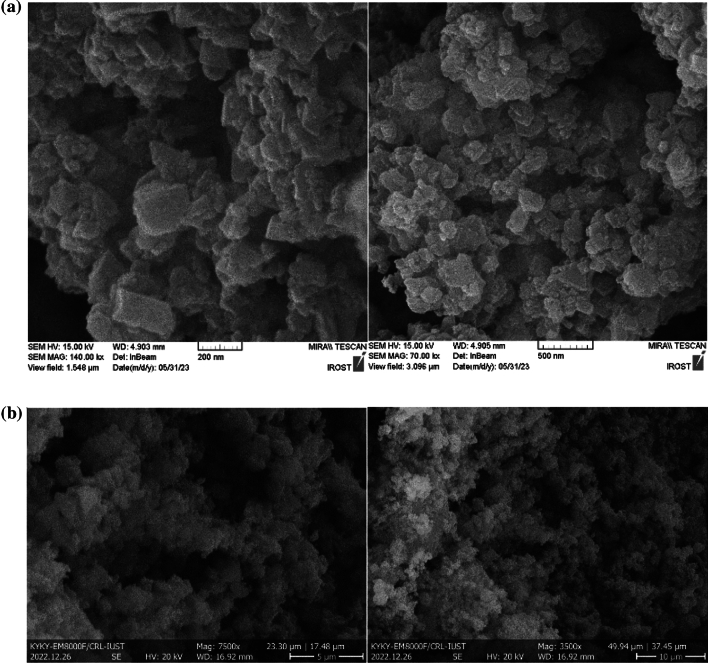
The SEM images of (**a**) MIL-100 and (**b**) MIL-100/Cell.

Figure 3 shows the SEM images of the MIL-100 and MIL-100/Cell. According to the obtained images, the approximately hexagonal structure of MIL-100 is proved. In addition, when we added cellulose and the MIL-100/Cell was synthesized, the MIL-100 particles dispersed very well on the surface of the cellulose, and SEM images show this dispersion clearly.

**Figure 4:**
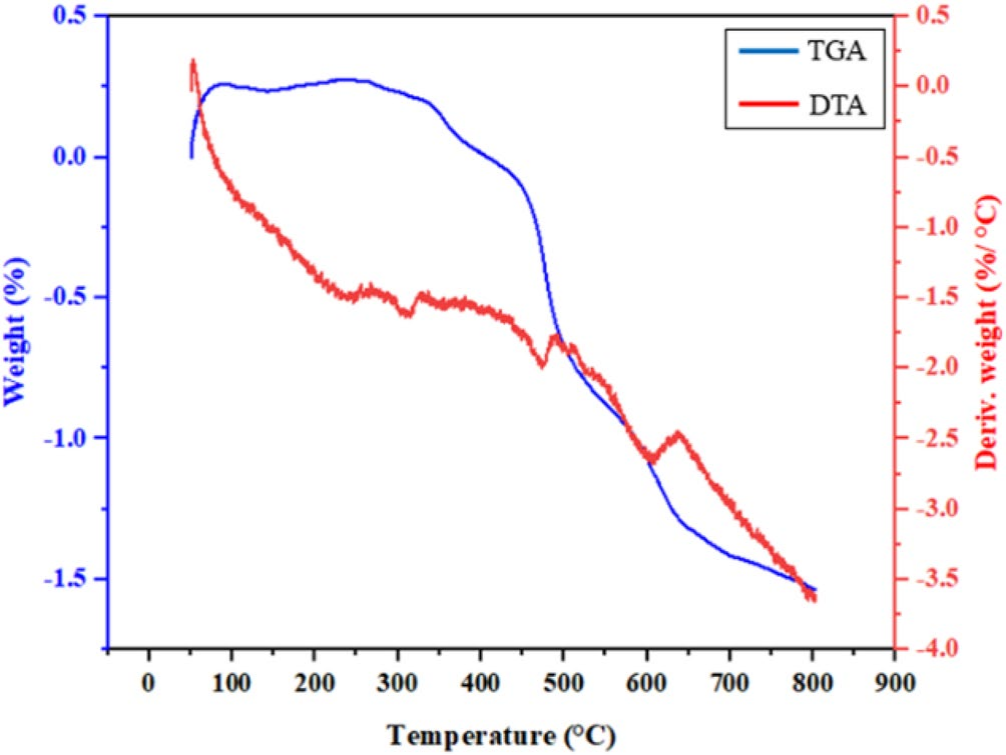
TGA/DTA analysis of the MIL-100/Cell.

The TGA/DTA analysis was used to investigate the thermal stability of the MIL-100/Cell (Fig. 4). The first stage of thermal degradation is 50–250 °C related to the removal of water molecules trapped in the porous structure of the MIL-100/Cell. The loss of weight below 250 °C is related to the removal of the water as well as ethanol which is used for washing the MOF. At the temperature of 250–350 °C, the cellulose in the structure is destroyed. It means that at this stage the cellulose was almost completely burnt owing to oxidative thermal degradation and also the crystal structure of the cellulose was destroyed. The weight loss at 350–400 °C is due to the collapse of the MIL-100 structure owing to the decomposition of the BTC ligand. The subsequent weight loss at 400–600 °C is due to the continued decomposition of MIL-100 along with iron reduction. In addition, in this stage, the cellulose is completely burned as well as oxidation of all the carbon and hydrogens of the organic molecule occurs and CO_2_ and H_2_O are formed. The destruction of the MOF structure takes place in the temperature range of 600–800 °C.

**Figure 5:**
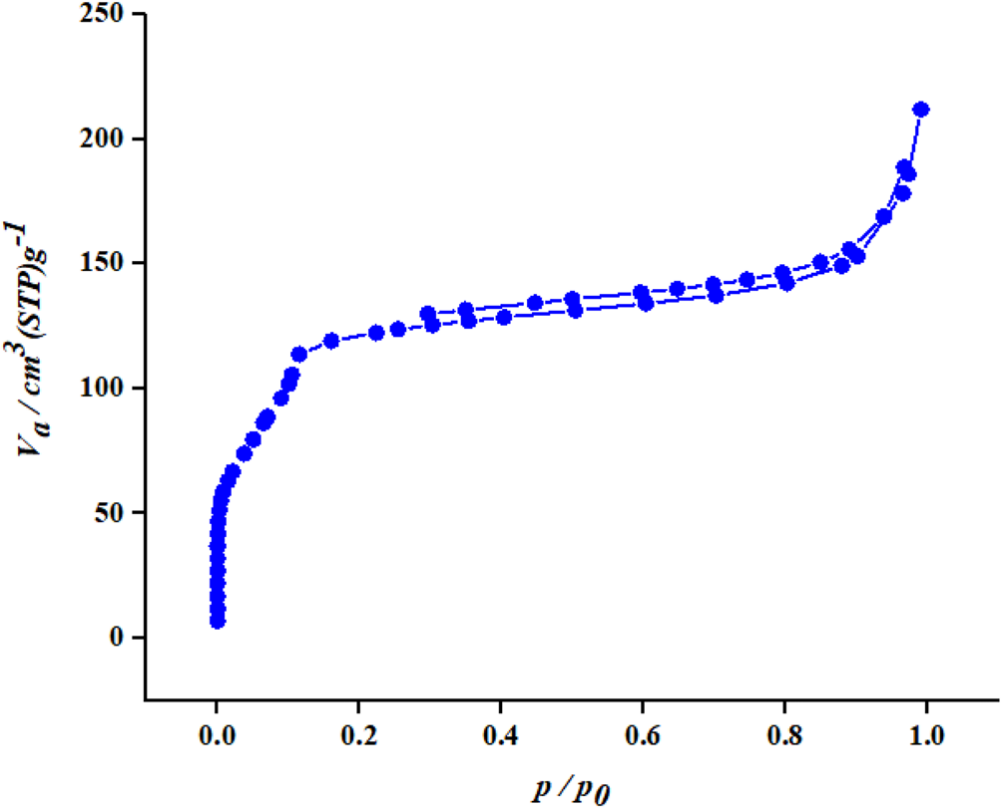
Nitrogen adsorption/desorption isotherm.

The nitrogen adsorption/desorption isotherm of MIL-100/Cell is according to Fig. 5. The results of the BET analysis can be seen in Table 1. The BET isotherm corresponds to the type п isotherm, which corresponds to porous materials.

**Table 1:** The results of BET analysis.

MOF	BET (m^2^/g)	Pore volume (cm^3^/g)	Mean pore diameter (nm)
MIL-100/Cell	294	0.3251	4.4194

**Figure 6:**
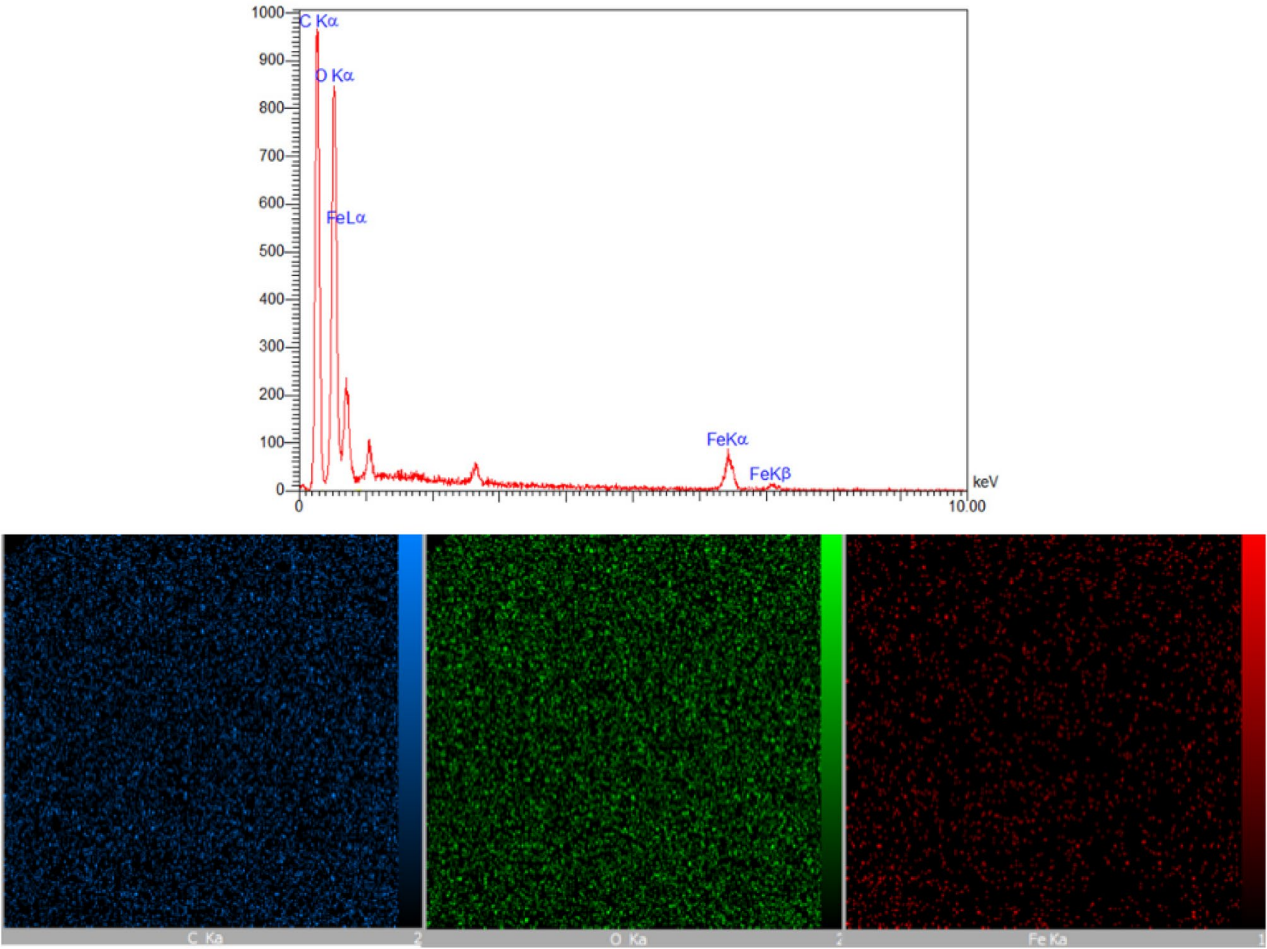
The EDS and elemental mapping analysis of the MIL-100/Cell.

The existence of C, O and Fe elements in the MIL-100/Cell structure was confirmed by EDS and elemental mapping according to Fig. 6. These analyses displayed that the MIL-100/Cell was synthesized.

In addition, we analyzed the charge of the MIL-100/Cell using zeta potential analysis. The result of the zeta potential displayed the − 3.5 mV in the aqueous phase at pH 7. This result displayed that the MIL-100/Cell has negative charge.

### Adsorption studies

Factors such as the dye concentration, amount of adsorbent, pH, and contact time are effective in the adsorption process.

#### Effect of MB concentration

**Figure 7:**
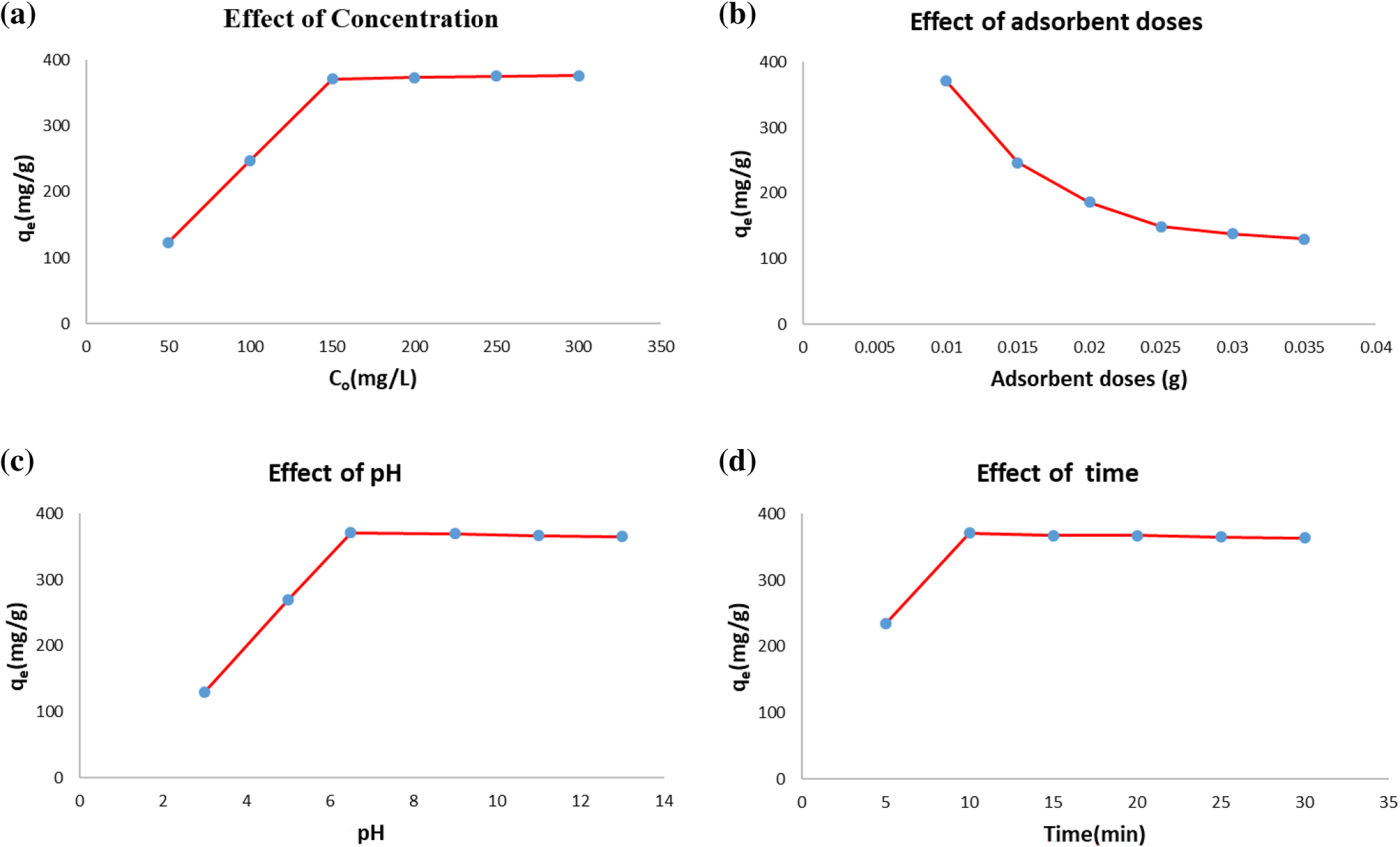
Effect of (**a**) Concentration (**b**) adsorbent dose (**c**) pH (C_o_ = 150 mg/L, adsorption dose = 0.01g, time = 10 min) and d) time (C_o_ = 150 mg/L, adsorption dose = 0.01g, pH = 6.5), on adsorption capacity of MB by MIL-100/Cell.

One of the most significant and influential factors in the adsorption capacity is the initial dye concentration. To examine the effect of initial dye concentration in the adsorption procedure, we chose different concentrations of MB including 50, 100, 150, 200, 250, and 300 (mg/L), and their adsorption capacity Ca was measured in the presence of 10 mg of MIL-100/Cell (Fig. 7a). As the dye concentration increases from 50 to 150 (mg/g), the adsorption capacity increases and with further increase remains constant.

#### Effect of adsorbent dose

Figure 7b displays the adsorption capacity for different amounts of MIL-100/Cell. According to the achieved results, the highest amount of adsorption capacity occurs in the presence of 0.01 g of MIL-100/Cell.

#### Effect of pH

Figure 7c displayed the adsorption capacity of MIL-100/Cell through changes in pH from 3 to 13. Experiments exhibited that by increasing the pH from 3 to 6.5 the adsorption capacity increased. As the pH increases, the adsorbent surface becomes negatively charged and facilitates electrostatic interaction with cationic dye molecules. In the range of 6.5 to 13 the pH remains constant and does not change much, which indicates that electrostatic interaction is not the only mechanism of dye adsorption^36^.

#### Effect of contact time

Figure 7d displays the effect of contact time on adsorption capacity. By increasing the contact time up to 10 min, the adsorption capacity increases and reaches its maximum value in 10 min. The quick increase in adsorption capacity at the start of the procedure is owing to the accessibility of most of the active sites for the adsorption of dye molecules. Active sites gradually fill up over time, which slows down adsorption^36^.

### Adsorption isotherm studies

**Figure 8:**
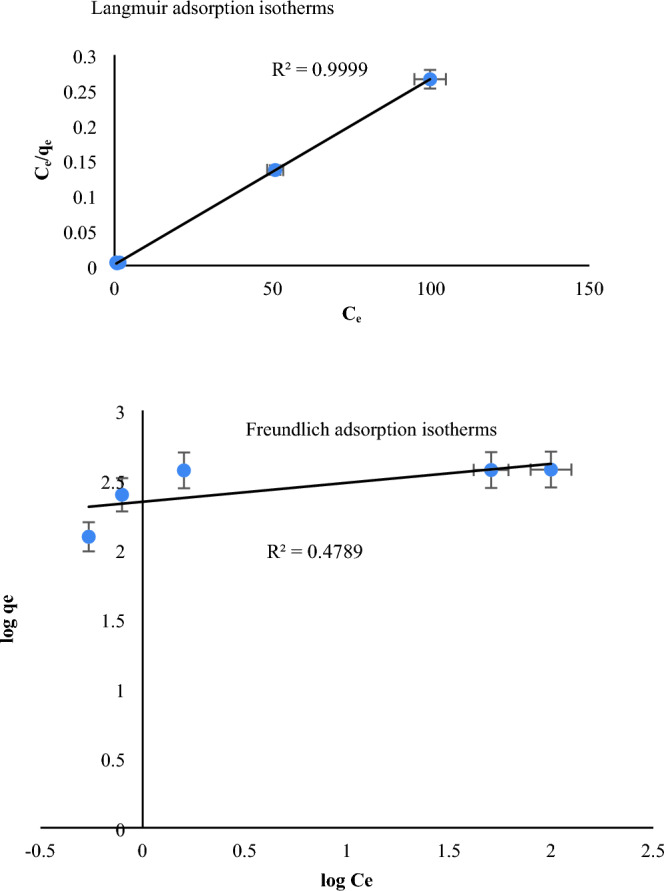
Langmuir and Freundlich adsorption isotherms.

Equilibrium data, commonly recognized as adsorption isotherms, are applied to define the adsorption mechanism. The adsorption isotherms describe how the adsorbed molecules or ions interact with the adsorbent surface^37–42^. In this work, Langmuir and Freundlich adsorption isotherms were applied to investigate the adsorption of MB dye by MIL-100/Cell using Eqs. (1) and (2):

1 $$\frac{{\text{C}}_{\text{e}}}{{\text{q}}_{\text{e}}}= \frac{1}{{\text{KLq}}_{\text{m}}}+\frac{{\text{C}}_{\text{e}}}{{\text{q}}_{\text{m}}}$$

2 $$\text{log }{\text{q}}_{\text{e}} =\text{ log }{\text{K}}_{\text{F}} +\frac{1}{\text{n}}\text{log }{\text{C}}_{\text{e}}$$

where q_m_ (mg/g) is the maximum adsorption capacity, q_e_ (mg/g) is equilibrium adsorption capacity, K_L_ is Langmuir adsorption constant, K_F_ is Freundlich constant and n indicates the adsorption intensity. The results of the data can be seen in Fig. 8 and Table 2. According to the correlation coefficients (R^2^) values obtained from the isotherm models, the MB adsorption process by MIL-100/Cell is more consistent with the Langmuir model (R^2^ = 0.999) and adsorption occurs monolayer. In other words, according to the results, R^2^ of the Langmuir isotherm model linear fitting is nearly 1.00, while those of the Freundlich model linear fitting is under 0.90, which proposes that the adsorption isotherms satisfy the Langmuir model. The Langmuir adsorption isotherm model describes the monolayer adsorption of MB dye molecules on MIL-100/Cell adsorbent.

**Table 2:** The adsorption isotherms factors from Langmuir and Freundlich model.

Isotherm	Parameters		
Langmuir	R^2^ = 0.999	q_max_ (mg/g) = 384.6 RL = 0.004	K_L_ (L/mg) = 1.86
Freundlich	R^2^ = 0.479	n = 7.3	K_F_ (mg^1−n^. L^n^/g) = 2.34

The essential characteristic of the Langmuir isotherm can be conveyed in terms of a dimensionless constant called separation factor (RL, also called equilibrium parameter) which is definite by the following equation:

3 $$ {1}/{1} + {\text{K}}_{{\text{L}}} *{\text{C}}_{0} , $$

where C_0_ (mg/L) is the initial dye concentration and K_L_ (L/mg) is the Langmuir constant. The value of RL indicates the shape of the isotherms to be either unfavorable (RL > 1), linear (RL = 1), favorable (0 < RL < 1) or irreversible (RL = 0). According to result related of RL that indicated in Table 2, the isotherm is favorable because the amount of RL is between 0 and 1.

### Thermodynamics of dye adsorption

In order to examine the effect of temperature on the MB adsorption by MIL-100/Cell, Gibbs free energy (ΔG°), enthalpy changes (ΔH°) and entropy changes (ΔS°) were investigated by the following Eqs:

4 $$\text{ln }{\text{K}}_{\text{d}} =\frac{\Delta \text{S}^\circ }{\text{R}}-\frac{\Delta \text{H}^\circ }{\text{RT}}$$

5 $$ \Delta {\text{G}}^\circ \, = \, - {\text{RT ln K}}_{{\text{d}}} $$

6 $${\text{K}}_{\text{d}} =\frac{{\text{q}}_{\text{e}}}{{\text{C}}_{\text{e}}}$$

In these equations, R is the global gas constant (8.314 J mol^−1^K^−1^), T is the temperature (K) and K_d_ is the equilibrium constant. The results of thermodynamic calculations can be seen in Table 3.

**Table 3:** Results of thermodynamic calculations.

T(K)	ΔG°(J mol^−1^)	ΔH°(J mol^−1^)	ΔS°(J mol^−1^K^−1^)
293	− 13,749.227	− 10,769.124	10.171
313	− 13,952.647	− 10,769.124	10.171
333	− 14,156.067	− 10,769.124	10.171
353	− 14,359.487	− 10,769.124	10.171
373	− 14,562.907	− 10,769.124	10.171

The obtained results show that the amount of adsorption decreases with increasing temperature, so the adsorption of MB is physically by MIL-100/Cell. The negativity of ΔG° at all temperatures indicates the spontaneity of the MB adsorption procedure. In fact, due to the negative ΔG°, it means that the absorption of MB is spontaneous nature and feasible. The reduction in ΔG° with increasing temperature indicated that adsorption at high temperature is more appropriate. The negativity of ΔH° indicates the exothermic nature of the process and the positivity of ΔS° shows an increase in disorder of the system through the adsorption process. The positive values of ΔS° may be owing to some structural changes in the adsorbate and adsorbents through the adsorption procedure from aqueous solution onto the adsorbents. Also, positive value of ΔS° shows the enhancing randomness at the solid–liquid interface through the adsorption of MB on the adsorbents^43,44^.

## Mechanism of MB adsorption

**Figure 9:**
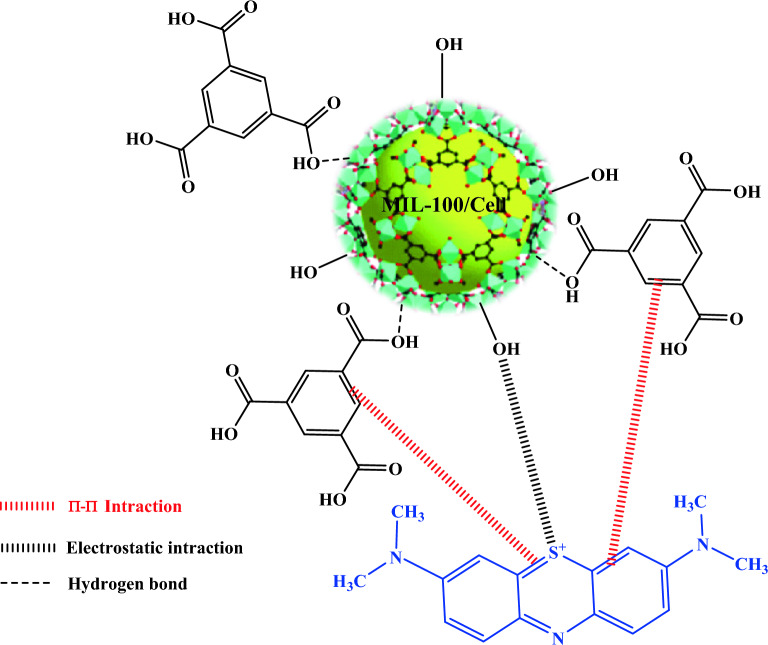
The suggested mechanism of MB adsorption by MIL-100/Cell.

The adsorption of MB on the MIL-100/Cell was occurring through electrostatic attraction and π-π stacking. The MB is a cationic organic dye and as a result on the surface of this dye, there are positive charges. On the other hand, the MIL-100/Cell has many electron pairs in its structure. In fact, according to the zeta potential analysis the MIL-100/Cell has − 3.5 mV and displayed the negative charge of the synthesized MOF. So when the MB was attracted on the surface of the MIL-100/Cell, the electrostatic attraction can occur between MB and MIL-100/Cell. In addition, in the chemical structure of the MB and MIL-100/Cell, some aromatic rings can be connected during π-π stacking attraction^45–48^. All the mentioned explanation is presented in Fig. 9.

## Comparison of MIL-100/Cell with other adsorbents

The comparison of the efficiency and adsorption performance of MIL-100/Cell with previously reported adsorbent for the adsorption of MB is tabulated in Table 4. As can be seen, MIL-100/Cell adsorbent exhibits higher adsorption performance in the removal of MB compared to the other adsorbent. In fact, in this work, we used a high concentration of the MB and at least the amount of the adsorbent. Compared to other works, according to the amount of MB concentration, pH, and catalyst amount, the time used for the reduction reaction has been very appropriate.

**Table 4:** Comparison of adsorption performance of the MIL-100/Cell with other adsorbent.

Adsorbent	q_max_ (mg/g) for MB	Operating conditions	References
MIL-101(Cr)	22	0.02 g adsorbent, 30 mg/L MB, 295 K	^49^
Fe_3_O_4_@MIL-100(Fe)	73.8	pH = 2–9, 60 mg/L MB, 318 K	^50^
MIL-53(Al)-NH_2_	167	5 mg/L MB, 10 mg adsorbent, pH = 2–7	^51^
MOF-235	187	25 °C, 40 mg/L MB, pH = 3–12	^52^
MIL-101-SO_3_H	351	298 K, pH = 3–12, 250 mg/L MB	^53^
MIL-100(Fe)-SBA-15	38	25 mg adsorbent, 50 mg/L MB, 25 °C, pH = 6	^54^
MIL-100/Cell	384.615	150 mg/L MB, pH = 6.5, 10 mg adsorbent, room temperature	This work

## Recycling of the MIL-100/Cell

**Figure 10:**
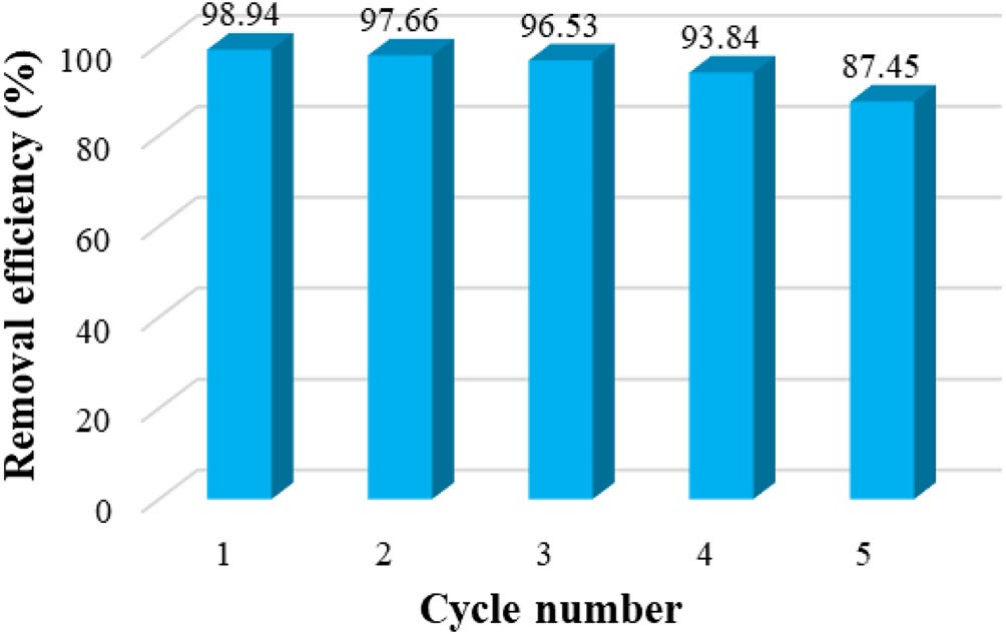
The RE (%) of MB by MIL-100/Cell after 5 cycles**.**

**Figure 11:**
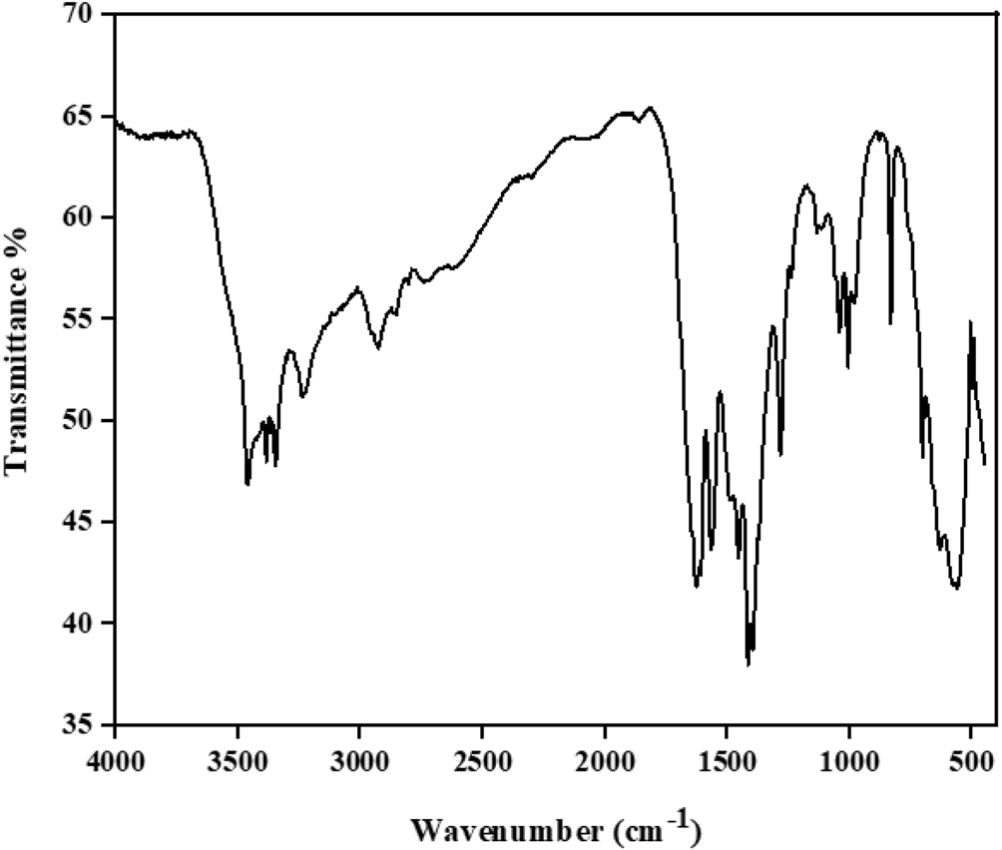
The FTIR analysis of the recycled MIL-100/Cell.

When the adsorption of the MB was finished the MIL-100/Cell adsorbent was washed with ethanol after collected by centrifugation and dried at 70 C° until reused in the next cycle. The results show that the dye RE is almost constant up to 3 cycles and after that, it decreases at a slow rate Fig. 10. The adsorbent can be recycled 5 times without noteworthy loss of performance. We analyzed the reused MIL-100/Cell adsorbent with FTIR analysis (Fig. 11). According to the FTIR analysis, the bonds in the recyclable adsorbent was preserved and this is mean that the structure of the adsorbent was kept. The peaks at 901 and 1063 cm^-1^ correspond to the β-glycosidic bond and –C–O–C– pyranose ring, respectively. Also the peaks at 1374 and 2900 cm^-1^ are correlated to the stretching and bending vibrations of –CH of glucose units of cellulose. In addition, the peaks at 615 and 1620 cm^-1^ are related to the C=O in the structure of the BTC and Fe–O respectively. The C=C bond of the benzene ring at 1379 and 1446 cm^−1^, and the –CH of benzene at 712 and 763 cm^−1^ are confirmed. these results displayed the structure of the adsorbent was remaining because the structure of the adsorbent is porous, and during dye adsorption, the dye is placed inside the adsorbent cavities, and as a result of washing, it is removed from the cavities, and as a result, the adsorbent structure is not destroyed.

## Conclusion

Dyes are among the most released industrial effluents into the environment. The removal of organic dyes is a global challenge due to the occurrence of various diseases in humans. This study demonstrated the effectiveness of MIL-100/Cell in removing cationic MB dye from an aqueous medium. The MIL-100/Cell was synthesized by a simple and green method at room temperature using water as a green solvent. The influence of parameters related to operating conditions such as contact time, amount of adsorbent, pH, and initial dye concentration were examined. According to the optimum reaction condition we used 10 mg of the MIL-100/Cell that can remove 98.94% of MB dye the initial concentration, time, pH and temperature are 150 mg/L, 10 min, 6.5, and room temperature respectively. The plot of the adsorption isotherms shows that the Langmuir model perfectly represents the adsorption of MB on MIL-100/Cell with a maximum adsorption capacity of 384.6 mg/g after 10 min. The thermodynamic parameters obtained indicate that the adsorption of MB dye on MIL-100/Cell is a spontaneous and exothermic process. The mechanism of MB adsorption proceeds through п-п and electrostatic interactions. According to the BET analysis the specific surface area of the synthesized MOF is 294 m^2^/g which related to the presence of cellulose as an efficient and green support. The MIL-100/Cell has many advantages such as a simple and green synthesis method, the use of inexpensive and non-toxic cellulose in the structure, and high adsorption capacity. The abundance of cellulose can offer a low-cost adsorption material that can potentially contribute to the treatment of textile effluents.

## Experimental section

### Materials and method

All materials applied in this study were bought from Merck and Aldrich Chemical Co. and applied without extra purification. The prepared adsorbent is characterized by different analytical methods like Fourier transform infrared (FTIR) (S8400 (Shimadzu Japan)), X-ray powder diffraction (XRD) (D8 advance (Bruker̦ Germany)), Scanning electron microscope (SEM) (EM8000 (KYKY, China)), Thermal gravimetric analysis (TGA/DTA) (STA504 (Bahr company̦ Germany)), Ehlers-Danlos syndromes (EDS) (TESCAN4992 (Brno̦ Czech Republic)), zeta potential analysis (Particlemetrix, ZETA-check (ZETA)) and Brunauer–Emmett–Teller (BET) (Belsorp mini ll (BEL company̦ Japan)).

### Synthesis of MIL-100/Cell

**Figure 12:**
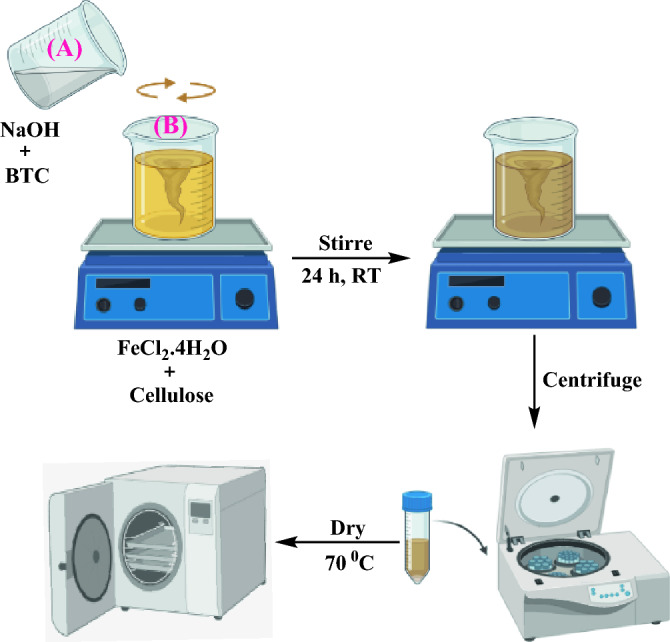
The synthesis procedure of MIL-100/Cell.

MIL-100(Fe) on cellulose was prepared according to a method from the literature^55^. First 1.676 g of benzene-1,3,5-tricarboxylic acid (BTC) was dissolved in sodium hydroxide (NaOH, 1 M, 30 mL) (Solution A). Then, FeCl_2_.4H_2_O (2.26 g) was dissolved in deionized water (97.2 mL) added dropwise to cellulose (300 mg), and stirred for 30 min at room temperature (Solution B). Solution A was added dropwise to solution B and stirred at ambient temperature for 24 h. The obtained brown product was filtered and washed with ethanol and dried at 70 °C for 24 h (MIL-100/Cell). The MIL-100/Cell was activated under vacuum at 120 °C for 6h (Fig. 12).

### Adsorption experiments

**Figure 13:**
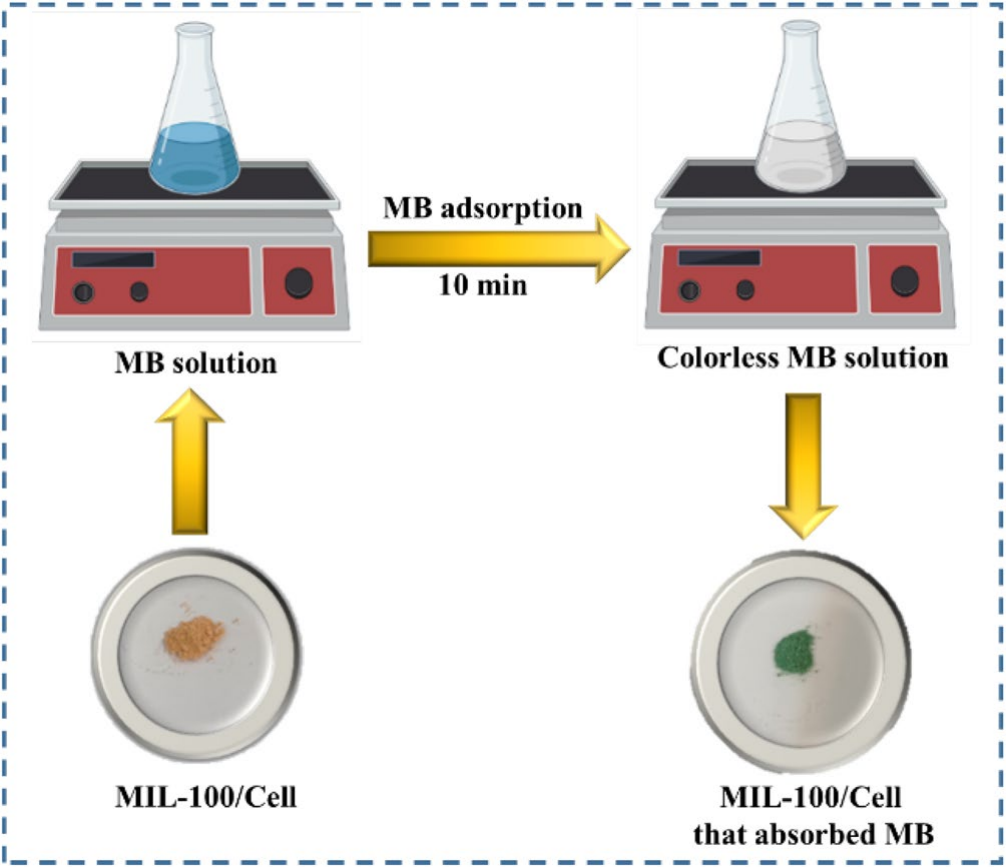
Adsorption of MB using MIL-100/Cell.

A standard solution of 250 mg/L of MB was prepared and used to prepare solutions of different concentrations by suitable dilution with double distilled water. The adsorption experiments were performed at room temperature and atmospheric pressure (Fig. 13). A solution of 50 mL MB (150 mg/L) was transferred into a 100 mL Erlenmeyer flask containing 10 mg of MIL-100/Cell and the mixture was magnetically stirred at constant speed (300 rpm). To assess the progress of the reaction, sampling was performed at different times of 5, 10, 15, 20, 25, and 30 min and the absorbance was characterized by a UV–vis spectrum at 663 nm. The calibration curve was obtained by plotting absorbance at the λ_max_ = 663nm. The removal efficiency (RE%), and adsorption capacity q_e_ (mg/g) of MIL-100/Cell were calculated according to the following equations (Eqs. (7) and (8)):

7 $$ {\text{RE }}\left( \% \right) \, = \, \left( {{\text{C}}_{0} - {\text{C}}_{t} } \right)/\left( {{\text{C}}_{0} } \right) \times {1}00, $$

8 $$ {\text{q}}_{{\text{e}}} = \, \left( {\left( {{\text{C}}_{0} - {\text{C}}_{t} } \right){\text{V}}} \right)/{\text{M,}} $$

where C_o_ and C_t_ (mg/L) are respectively the concentrations initial and at time *t*, V (L) is the volume of solution and M (g) is the mass of adsorbent.

#### Effect of concentration

For this purpose, we prepared MB solutions at various concentrations: 50, 100, 150, 200, 250, and 300 (mg/L). Subsequently, in adsorption tests conducted in a 100 mL beaker, 50 mL of each solution was combined with 10 mg of MIL-100/Cell. The resulting mixture underwent magnetic stirring at a consistent speed (300 rpm) under room temperature and neutral pH conditions. Following a 10-min interval, the adsorbent was isolated via centrifugation, and the solution was characterized using a UV–vis spectrum. The quantity q_e_ was determined using Eq. (2), and a corresponding plot is depicted in Fig. 7a.

#### Effect of adsorbent dose

In a previous experiment, it was established that the optimal MB concentration is 150 mg/L. In the current phase of our study, a solution precisely adhering to this concentration was prepared and 50 mL of it was introduced into a 100 mL vessel. Subsequently, varying quantities of MIL-100/Cell adsorbents, ranging from 10 to 35 mg, were added to the solution, which was then agitated at a constant speed of 300 rpm. This procedure was carried out at room temperature and under neutral pH conditions for a duration of 10 min. Following the separation of the adsorbent through centrifugation, the solution was analyzed using a UV–vis spectrum. By applying Eq. (8), the value of q_e_ was determined and the results were depicted in Fig. 7b.

#### Effect of pH

In previous tests, we choose 150 mg/L and 10 mg of MB concentration and dose of adsorbent respectively. In this step we added 50 mL of MB solution with 150 mg/L concentration and added 10 mg of adsorbent and the mixture was magnetically stirred at constant speed (300 rpm) at room temperature in different pH including acidic and basic. We used a solution of HCl (0.1 M) and NaOH (0.1 M) to make the solution acidic and basic. For this aim, we controlled the pH of the solution using pH meter and we performed the reaction at different pH including 3, 5, 6.5, 9, 11, 13. After 10 min, the adsorbent was separated using a centrifuge and a UV–vis spectrum characterized the solution. We calculated qe using Eq. (2) and the plot was drawn that is shown in Fig. 7c.

#### Effect of contact time

In our earlier experiments, we utilized a solution containing 150 mg/L MB concentration, 10 mg of adsorbent, and pH = 6.5 to evaluate the adsorption performance. In the current phase, we endeavor to explore the impact of different contact times on the adsorption performance. To accomplish this, we introduced 50 mL of the MB solution (150 mg/L) and 10 mg of adsorbent to a 100 mL beaker, adjusting the pH to 6.5. We then subjected the mixture to room temperature and varied the reaction times to 10, 15, 20, 25, and 30 min. At the end of each reaction time, we isolated the adsorbent via centrifugation and analyzed the solution using UV–vis spectrometry. We calculated q_e_ utilizing Eq. (2) and graphed the outcome, as depicted in Fig. 7d.

## Data Availability

All data generated or analysed during this study are included in this published article.
